# A Proposal for Practical Diagnosis of Renal Hypouricemia: Evidenced from Genetic Studies of Nonfunctional Variants of *URAT1/SLC22A12* among 30,685 Japanese Individuals

**DOI:** 10.3390/biomedicines9081012

**Published:** 2021-08-13

**Authors:** Yusuke Kawamura, Akiyoshi Nakayama, Seiko Shimizu, Yu Toyoda, Yuichiro Nishida, Asahi Hishida, Sakurako Katsuura-Kamano, Kenichi Shibuya, Takashi Tamura, Makoto Kawaguchi, Satoko Suzuki, Satoko Iwasawa, Hiroshi Nakashima, Rie Ibusuki, Hirokazu Uemura, Megumi Hara, Kenji Takeuchi, Tappei Takada, Masashi Tsunoda, Kokichi Arisawa, Toshiro Takezaki, Keitaro Tanaka, Kimiyoshi Ichida, Kenji Wakai, Nariyoshi Shinomiya, Hirotaka Matsuo

**Affiliations:** 1 Department of Integrative Physiology and Bio-Nano Medicine, National Defense Medical College, Tokorozawa 359-8513, Japan; ykawamura@ndmc.ac.jp (Y.K.); aknak@ndmc.ac.jp (A.N.); s.shimizu11@gmail.com (S.S.); ytoyoda-tky@umin.ac.jp (Y.T.); makoto.kawaguchi.2920@gmail.com (M.K.); shinomi@ndmc.ac.jp (N.S.); 2 Department of Pharmacy, Faculty of Medicine, The University of Tokyo Hospital, The University of Tokyo, Tokyo 113-8655, Japan; tappei-tky@umin.ac.jp; 3 Department of Preventive Medicine, Faculty of Medicine, Saga University, Saga 849-8501, Japan; ynishida@cc.saga-u.ac.jp (Y.N.); harameg@cc.saga-u.ac.jp (M.H.); tanakake@cc.saga-u.ac.jp (K.T.); 4 Department of Preventive Medicine, Graduate School of Medicine, Nagoya University, Nagoya 466-8550, Japan; a-hishi@med.nagoya-u.ac.jp (A.H.); ttamura@med.nagoya-u.ac.jp (T.T.); k.takeuchi@med.nagoya-u.ac.jp (K.T.); wakai@med.nagoya-u.ac.jp (K.W.); 5 Department of Preventive Medicine, Graduate School of Biomedical Sciences, Tokushima University, Tokushima 770-8503, Japan; skamano@tokushima-u.ac.jp (S.K.-K.); karisawa@tokushima-u.ac.jp (K.A.); 6 Department of International Island and Community Medicine, Graduate School of Medical and Dental Sciences, Kagoshima University, Kagoshima 890-8544, Japan; shibuya.kenichi@gmail.com (K.S.); iburie@m2.kufm.kagoshima-u.ac.jp (R.I.); takezaki@m.kufm.kagoshima-u.ac.jp (T.T.); 7 Department of Emergency and Intensive Care Medicine, Graduate School of Medical and Dental Sciences, Kagoshima University, Kagoshima 890-8544, Japan; 8 Department of Preventive Medicine and Public Health, National Defense Medical College, Tokorozawa 359-8513, Japan; s-suzuki@ndmc.ac.jp (S.S.); iwasawa@ndmc.ac.jp (S.I.); hnakashi@ndmc.ac.jp (H.N.); mtsunoda@ndmc.ac.jp (M.T.); 9 Department of Health and Welfare System, College of Nursing Art and Science, University of Hyogo, Akashi 673-8588, Japan; hirokazu_uemura@cnas.u-hyogo.ac.jp; 10 Department of Pathophysiology, Tokyo University of Pharmacy and Life Sciences, Tokyo 192-0392, Japan; ichida@toyaku.ac.jp; 11 Division of Kidney and Hypertension, Department of Internal Medicine, Jikei University School of Medicine, Tokyo 105-8461, Japan

**Keywords:** *URAT1/SLC22A12*, renal hypouricemia (RHUC), serum uric acid (SUA), fractional excretion of uric acid (FE_UA_)

## Abstract

Background: Renal hypouricemia (RHUC) is characterized by a low serum uric acid (SUA) level and high fractional excretion of uric acid (FE_UA_). Further studies on FE_UA_ in hypouricemic individuals are needed for a more accurate diagnosis of RHUC. Methods: In 30,685 Japanese health-examination participants, we genotyped the two most common nonfunctional variants of *URAT1* (NFV-*URAT1*), W258X (rs121907892) and R90H (rs121907896), in 1040 hypouricemic individuals (SUA ≤ 3.0 mg/dL) and 2240 individuals with FE_UA_ data. The effects of NFV-*URAT1* on FE_UA_ and SUA were also investigated using linear and multiple regression analyses. Results: Frequency of hypouricemic individuals (SUA ≤ 3.0 mg/dL) was 0.97% (male) and 6.94% (female) among 30,685 participants. High frequencies of those having at least one allele of NFV-*URAT1* were observed in 1040 hypouricemic individuals. Furthermore, NFV-*URAT1* significantly increased FE_UA_ and decreased SUA, enabling FE_UA_ and SUA levels to be estimated. Conversely, FE_UA_ and SUA data of hypouricemic individuals are revealed to be useful to predict the number of NFV-*URAT1*. Conclusions: Our findings reveal that specific patterns of FE_UA_ and SUA data assist with predicting the number of nonfunctional variants of causative genes for RHUC, and can also be useful for practical diagnosis of RHUC even before genetic tests.

## 1. Introduction

Renal hypouricemia (RHUC), an overexcretion-type hypouricemia, is an inherited disorder caused by increased urinary urate excretion that results from insufficient renal urate reabsorption [1]. Dysfunctions in urate transporter 1 (URAT1) [2] and glucose transporter 9 (GLUT9) [3] respectively, cause RHUC type 1 and 2, showing low serum uric acid (SUA) levels and high fractional excretion of uric acid (FE_UA_). Although most hypouricemia patients are normally asymptomatic and are found by chance in health examinations, RHUC is sometimes accompanied by severe complications, such as exercise-induced acute kidney injury (EIAKI) and urolithiasis [4,5].

Several urate transporters play an important physiological role in urate handling by urate excretion and reabsorption from the human kidney. *SLC22A12/URAT1* is a causative gene for RHUC type 1 [2], and its nonfunctional variants of *URAT1* (NFV-*URAT1*), W258X (rs121907892) and R90H (rs121907896), are reportedly the two most common causative variants in the Japanese population [6,7].

Several studies have previously reported the distribution of SUA levels in large Japanese populations [3,8,9,10]. Although the frequency of NFV-*URAT1* is relatively high in Japanese people, the frequency of NFV-*URAT1* in those with lower SUA (≤3.0 mg/dL) has not been studied in Japan or elsewhere. Furthermore, the effect size on FE_UA_ of the number of alleles for NFV-*URAT1* has never been clarified.

This state of affairs prompted us to investigate, in this study, the frequency of NFV-*URAT1* in 1040 hypouricemic individuals (SUA ≤ 3.0 mg/dL) among 30,685 Japanese individuals undergoing health examinations. Using genetic analyses of these Japanese individuals, we evaluated the effect of NFV-*URAT1* on FE_UA_ with the aim of being able to predict the presence and number of NFV-*URAT1* from their FE_UA_ and SUA levels. This should lead to a more practical diagnosis of RHUC from patients’ laboratory data.

## 2. Materials and Methods

### 2.1. Study Participants

This study was approved by the National Defense Medical College and Nagoya University’s institutional ethical committees. We performed all the processes in accordance with the Declaration of Helsinki.

All the 30,685 Japanese participants (13,607 males and 17,078 females) in this study were recruited from participants in health examinations in the Shizuoka, Daiko (Aichi), Tokushima, Saga and Kagoshima areas in the Japan Multi-Institutional Collaborative Cohort Study (J-MICC Study) [11,12]. Written informed consent was obtained from each participant.

Those with low SUA of ≤3.0 mg/dL were defined as “hypouricemic individuals”. Among them, those with SUA ≤ 2.0 mg/dL and 2.0 mg/dL < SUA ≤ 3.0 mg/dL were defined as “hypouricemia” and “mild hypouricemia”, respectively. Hypouricemia was further divided into two groups: “severe hypouricemia” (SUA ≤ 1.0 mg/dL) and “moderate hypouricemia” (1.0 < SUA ≤ 2.0 mg/dL). When available, FE_UA_ was calculated from the results of blood and urine tests using the equation: [urinary uric acid (mg/dL) × serum creatinine (mg/dL)]/[SUA (mg/dL) × urinary creatinine (mg/dL)] [1,13].

### 2.2. Genetics Analysis

The genomic DNA of each participant was extracted from whole peripheral blood cells. For genotyping, we performed the TaqMan method (Life Technologies, Carlsbad, CA, USA) using a LightCycler 480 (Roche Diagnostics, Mannheim, Germany), as described previously [7]. For NFV-*URAT1*, we genotyped the two most common variants (W258X and R90H). We used custom TaqMan assay probes designed for R90H, VIC-CCGCCACTTCCGC and FAM-CGCCGCTTCCGC, and for W258X, VIC-CGGGACTGAACACTG and FAM-CGGGACTGGACACTG. Direct sequencing was performed with a 3130xl Genetic Analyzer (Life Technologies) to confirm all the heterozygotes and homozygotes of NFV-*URAT1*, using the following primers [7]: for R90H, forward 5′-GTTGGAGCCACCCCAAGTGAC-3′ and reverse 5′-GTCTGACCCACCGTGATCCATG-3′; for W258X, forward 5′-TGATGAACACGGGCACTCTC-3′ and reverse 5′-CTTTCCACTCGCTCCCCTAG-3′.

### 2.3. Data Analysis

Linear regression analyses were performed to evaluate the influence of the allele of NFV-*URAT1* on FE_UA_ or SUA. We also carried out multiple regression analysis in a stepwise method using the following equation: y  =  β_0_  +   β_1_x_1_  +  β_2_x_2_, where y is FE_UA_ or SUA levels, x_1_ is a dummy variable representing whether the number of alleles of NFV-*URAT1* is one (one allele  =  1 and other  =  0), x_2_ is a dummy variable representing whether the number of alleles of NFV-*URAT1* is two (two alleles  =  1 and other  =  0) and β_0_, β_1_ and β_2_ are partial regression coefficients for each covariate. We used SPSS v. 22 (IBM Japan, Tokyo, Japan) for all calculations in the statistical analyses carried out in this study.

## 3. Results

### 3.1. Distribution of SUA Levels in the Japanese Population

Table 1 shows the distribution of SUA levels of the 30,685 Japanese health examination participants (13,607 males and 17,078 females). Among the 30,685 participants, the prevalence of hypouricemia (SUA ≤ 2.0 mg/dL) was 0.18% in males and 0.54% in females. Mild hypouricemia (2.0 < SUA ≤ 3.0 mg/dL) was observed in 107 males (0.79%) and 1093 females (6.40%). Hypouricemic individuals (SUA ≤ 3.0 mg/dL) consisted of 131 males (0.97%) and 1186 females (6.94%). The frequency of moderate hypouricemia in males (1.0 < SUA ≤ 2.0 mg/dL) was 0.03%, the fewest in all male participants according to the ranked classification of SUA used in this study (Table 1). Contrary to the pattern seen in hypouricemic individuals (SUA ≤ 3.0 mg/dL), the frequency of hyperuricemia (SUA > 7.0 mg/dL) was 20.28% for males and 1.11% for females.

### 3.2. Frequency of NFV-URAT1 in Hypouricemic Individuals

**Figure 1:**
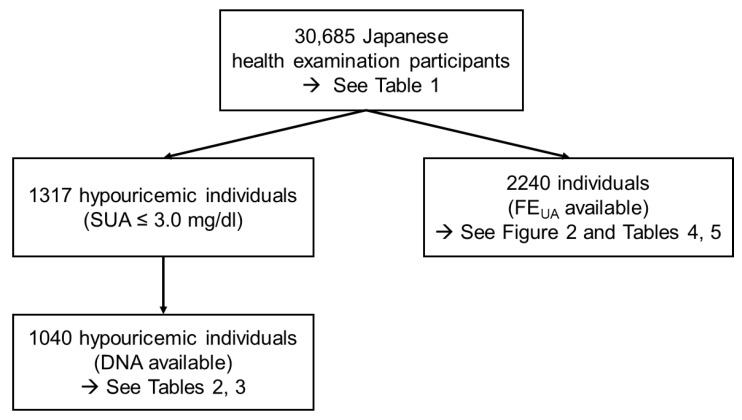
Selection of participants for each analysis. We first collected SUA data from 30,685 Japanese participants (13,607 males and 17,078 females) to gain an understanding of the distribution of SUA levels in the Japanese general population. Second, to investigate the frequency of NFV-*URAT1* alleles, 1040 hypouricemic individuals (108 males and 932 females) with SUA of ≤3.0 mg/dL and whose genomic DNA samples were available were selected from 1317 hypouricemic individuals. Third, to evaluate the relationship between NFV-*URAT1* and FE_UA_ or SUA, 2240 individuals (1542 males and 698 females) whose FE_UA_ data were available were also selected from all 30,685 participants. SUA, serum uric acid; FE_UA_, fractional excretion of uric acid; NFV-*URAT1*, nonfunctional variants of *URAT1*.

As displayed in Figure 1, 1040 hypouricemic individuals (SUA ≤ 3.0 mg/dL) were selected from the whole population of participants to investigate the frequency of NFV-*URAT1.* The characteristics of these hypouricemic individuals (SUA ≤ 3.0 mg/dL) are shown in Table 2. Table 3 indicates the relationship between the number of NFV-*URAT1* and hypouricemic populations. As shown here, those with two NFV-*URAT1* alleles were seen only in severe hypouricemia in both sexes. For mild hypouricemia, the largest population was males with one NFV-*URAT1* allele, although it was those with 0 alleles in females. Of the mild hypouricemia individuals, at least two thirds of the males and one third of the females were assumed to be the “mild” RHUC type 1 due to having only one NFV-*URAT1* allele.

### 3.3. Associations between NFV-URAT1 and FE_UA_ or SUA in 2240 Japanese Individuals

**Figure 2:**
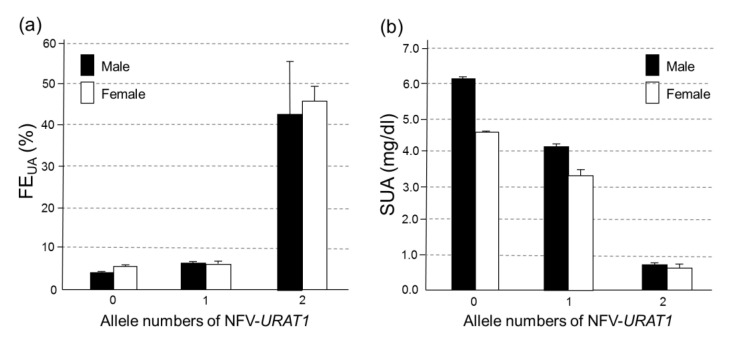
The effect on FE_UA_ and SUA levels of NFV-*URAT1* in 2240 Japanese participants: (**a**) FE_UA_ and (**b**) SUA levels of participants with 0, 1 and 2 alleles of NFV-*URAT1* are shown for each sex. Among the 1542 male participants (black bars), 1472, 68 and 2 participants had 0, 1 and 2 alleles of NFV-*URAT1*, respectively. Among the 698 female participants (white bars), 678, 18 and 2 participants had 0, 1 and 2 alleles of NFV-*URAT1*, respectively. The presence of NFV-*URAT1* alleles significantly increased FE_UA_ (*p* = 1.27 × 10^−46^ in males and *p* = 5.09 × 10^−27^ in females), and significantly decreased SUA (*p* = 2.47 × 10^−53^ in males and *p* = 2.14 × 10^−13^ in females). W258X and R90H, the two most common variants of *URAT1*, were selected as NFV-*URAT1* in this study. All bars are expressed as means  ±  SE. FE_UA_, fractional excretion of uric acid; SUA, serum uric acid; NFV-*URAT1*, nonfunctional variants of *URAT1.*

We evaluated the relationship between NFV-*URAT1* and FE_UA_ or SUA in 2240 Japanese individuals (Figure 1) whose FE_UA_ data were available. Figure 2 shows that, in both sexes, NFV-*URAT1* alleles significantly increased FE_UA_ (*p* = 1.27 × 10^−46^ in males and 5.09 × 10^−27^ in females) and decreased SUA (*p* = 2.47 × 10^−53^ in males and 2.14 × 10^−13^ in females). The mean FE_UA_ levels of those with 0, 1 and 2 alleles for NFV-*URAT1* were 3.94% ± 0.06%, 6.57% ± 0.39% and 42.6% ± 12.8% in males, and 5.37% ± 0.10%, 6.43% ± 0.67% and 45.9% ± 3.81% in females, respectively (Figure 2a). The mean SUA levels of those with 0, 1 and 2 alleles of NFV-*URAT1* were 6.10  ±  0.03 (mg/dL), 4.17 ± 0.11 (mg/dL) and 0.75 ± 0.04 (mg/dL) in males, and in females were 4.56 ±  0.04 (mg/dL), 3.31 ± 0.19 (mg/dL) and 0.65 ± 0.11 (mg/dL), respectively (Figure 2b).

Table 4 shows laboratory data including FE_UA_, SUA levels and NFV-*URAT1* of 52 hypouricemia and mild hypouricemia individuals (SUA ≤ 3.0 mg/dL) among those 2240 individuals whose FE_UA_ data were available (Figure 1). All four individuals (two males and two females) with two NFV-*URAT1* alleles showed high FE_UA_ (mean: 44.3%; range: 24.5–60.7%) and extremely low SUA levels of ≤1.0 mg/dL (severe hypouricemia). On the other hand, the other 48 individuals (6 males and 42 females) with one or no NFV-*URAT1* alleles displayed a mean FE_UA_ level of 8.01% (range: 2.05–16.86%). Most of them were mild hypouricemia (Table 4).

### 3.4. The Effect on FE_UA_ and SUA Levels of the Number of Alleles of NFV-URAT1

Table 5 shows the results of multiple regression analyses on FE_UA_ and SUA levels by the number of alleles of NFV-*URAT1*. Two alleles of NFV-*URAT1* (β_2_) markedly elevated FE_UA_ in both sexes by approximately 40% (β = 38.68, *p* = 1.35 × 10^−108^ for males, β = 40.54, *p* = 2.15 × 10^−79^ for females). One allele of NFV-*URAT1* (β_1_) gave significantly elevated FE_UA_ in males (β = 2.63, *p* = 4.04 × 10^−20^), but the variance of one allele of NFV-*URAT1* was eliminated for females in this multiple regression analysis. Conversely, two alleles of NFV-*URAT1* (β_2_) markedly reduced SUA levels (β = –5.35, *p* = 2.39 × 10^−12^ for male, β = –3.91, *p* = 2.97 × 10^−8^ for females). One allele of NFV-*URAT1* (β_1_) also significantly reduced SUA levels (β = –1.93, *p* = 6.56 × 10^−45^ for males, β = –1.25, *p* = 1.53 × 10^−7^ for females). In other words, these results indicate that FE_UA_ and SUA levels can be estimated from the genotyping results of NFV-*URAT1* (W258X and R90H), and, vice versa, from the clinical data (FE_UA_ and SUA levels), we can predict the presence and number of NFV-*URAT1*, which can reveal whether or not a patient is RHUC type 1.

## 4. Discussion

In this study, we demonstrated the prevalence of hypouricemia (SUA ≤ 2.0 mg/dL) and mild hypouricemia (2.0 < SUA ≤ 3.0 mg/dL) in a general Japanese population (Table 1), the frequency of NFV-*URAT1* in hypouricemic individuals (Table 3) and the effect of NFV-*URAT1* on FE_UA_ and SUA (Figure 2, Table 4; Table 5).

The prevalence of hypouricemia (SUA ≤ 2.0 mg/dL) was 0.18% for males and 0.54% for females in the present study (Table 1), which is consistent with previous reports and the clinical practice guideline for RHUC [1,8,10]. The prevalence of hypouricemia (SUA ≤ 2.0 mg/dL) in the previous report [1] was approximately 0.2% for males and 0.4% for females in the general Japanese population. The prevalence of mild hypouricemia (2.0 < SUA ≤ 3.0 mg/dL) and hypouricemic individuals (SUA ≤ 3.0 mg/dL) was also reported in the present study. The prevalence of moderate hypouricemia (1.0 < SUA ≤ 2.0 mg/dL) was 0.03% for males, the lowest among all the participants (Table 1), also consistent with previous reports [3,10].

Although the frequency of NFV-*URAT1* is high in Japanese [1] and European Roma populations [14,15], this is the first report on the frequencies of those having 0, 1 and 2 alleles of NFV-*URAT1* (W258X and R90H) in the general population of hypouricemia and mild hypouricemia individuals (SUA ≤ 3.0 mg/dL).

High frequencies of NFV-*URAT1* (in total 85.7% and 73.7% in males and females) are observed among hypouricemia (SUA ≤ 2.0 mg/dL; Table 3), suggesting that more than 70% of hypouricemic individuals (SUA ≤ 2.0 mg/dL) appear to have RHUC type 1 due to one or two NFV-*URAT1* alleles. Table 3 also indicates that even in mild hypouricemia (2.0 < SUA ≤ 3.0 mg/dL), at least two thirds of men and one third of women are estimated to be the “mild” RHUC type 1 due to the presence of only one NFV-*URAT1*. These results indicate that RHUC or “mild” RHUC should be suspected when examining hypouricemic individuals (SUA ≤ 3.0 mg/dL), as the clinical practice guideline for RHUC recommends in its clinical algorithm [1,16]. This study, however, was performed focusing solely on the two most common NFV-*URAT1* alleles (W258X and R90H). Further studies to identify other known dysfunctional variants [2,6,17,18,19,20] are needed, as well as the genotyping of novel variants of *URAT1* to be able to more accurately elucidate the frequency of RHUC type 1 in hypouricemic individuals (SUA ≤ 3.0 mg/dL).

We have for the first time demonstrated that NFV-*URAT1* significantly increases FE_UA_ and decreases SUA, using 2240 individuals whose FE_UA_ data were available (Figure 2). Furthermore, as shown in Table 5, we have proven that FE_UA_ and SUA levels can be estimated from the number of alleles of NFV-*URAT1* (W258X and R90H). We also suggest that it is possible to predict the presence and number of NFV-*URAT1* alleles from laboratory data (FE_UA_ and SUA levels). Of 52 hypouricemic individuals, 4 individuals (Cases Nos. 1–4 in Table 4), with 2 NFV-*URAT1* alleles, exhibited severe hypouricemia and high FE_UA_. On the other hand, most of the other 48 hypouricemic individuals (Case Nos. 5–52 in Table 4), with 1 or 0 NFV-*URAT1* alleles, exhibited mild hypouricemia and showed normal or slightly high FE_UA_. In other words, the high FE_UA_ that is seen in severe hypouricemia is a useful predictor of the presence of two NFV-*URAT1* alleles, and the normal or slightly high FE_UA_ seen in mild hypouricemia also helps to predict the presence of one or zero NFV-*URAT1* alleles.

The limitations of the present study are as follows: (1) menopausal status was not considered, and (2) the influence of environmental factors such as alcohol intake and medications were not adjusted. Further analyses will be necessary to elucidate the effects of these factors.

**Figure 3:**
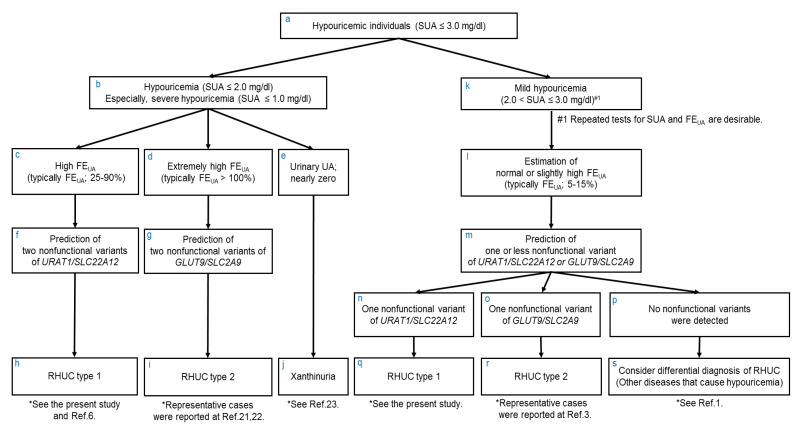
Flowchart to predict the number of nonfunctional variants of causative genes for RHUC based on FE_UA_ and SUA data. Based on the findings of the present study, together with previous reports, we hereby propose a method of making a more practical diagnosis of RHUC even before genetic testing for *URAT1/SLC22A12* or *GLUT9/SLC2A9*, when physicians detect and examine hypouricemic individuals (SUA ≤ 3.0 mg/dL; (**a**)). In those with hypouricemia (SUA ≤ 2.0 mg/dL), especially with severe hypouricemia (SUA ≤ 1.0 mg/dL) (**b**), their FE_UA_ and urinary UA data should be investigated (**c**–**e**). These data will help to estimate RHUC type 1 or 2 due to two nonfunctional variants of *URAT1* or *GLUT9*, or xanthinuria (**f**–**j**). Genetic analysis is needed to distinguish RHUC type 1 and type 2, but this flowchart shows that physicians should be able to predict the causative gene from patients’ laboratory data before performing a genetic analysis. On the other hand, in those with mild hypouricemia (2.0 < SUA ≤ 3.0 mg/dL; (**k**)), their FE_UA_ data are estimated to be normal or slightly high (**l**), which makes it possible to predict there to be one or no nonfunctional variants of *URAT1* or *GLUT9* (**m**). With these mild hypouricemia patients, detection of one nonfunctional variant of *URAT1* (**n**) or *GLUT9* (**o**) by genetic analysis is needed to make a diagnosis of RHUC type 1 (**q**) or type 2 (**r**). Physicians should consider differential diagnosis of RHUC (**s**) if no nonfunctional variants of *URAT1* or *GLUT9* are detected (**p**). Additionally, see Figure 4 regarding the patterns of FE_UA_ and SUA data of RHUC type 1 and type 2. SUA, serum uric acid; FE_UA_, fractional excretion of uric acid; UA, uric acid; RHUC, renal hypouricemia.

From these findings, together with previous reports [3,6,21,22], we hereby propose a more efficient method of diagnosis of RHUC based on FE_UA_ and SUA data (Figure 3), when physicians detect and examine hypouricemic individuals (SUA ≤ 3.0 mg/dL) (Figure 3a). With hypouricemia, especially severe hypouricemia (SUA ≤ 1.0 mg/dL) (Figure 3b), we propose the following three differential diagnoses (Figure 3c–j): (1) When the FE_UA_ data of severe hypouricemia patients are high (typically FE_UA_; 25–90%) (Figure 3c), these patients are predicted to be RHUC type 1 [6] (Figure 3h) because the laboratory data suggest that they should have two nonfunctional variants of *URAT1* (Figure 3f). (2) When the FE_UA_ data of severe hypouricemia patients are extremely high (typically FE_UA_ > 100%) (Figure 3d), these patients are predicted to be RHUC type 2 [21,22] (Figure 3i) because they are likely to have two nonfunctional variants of *GLUT9* (Figure 3g). (3) When the FE_UA_ data are not high and urinary uric acid (UA) levels are nearly zero in severe hypouricemia patients (Figure 3e), they are suspected of having xanthinuria [23] (Figure 3j). With mild hypouricemia (2.0 < SUA ≤ 3.0 mg/dL) (Figure 3k), their FE_UA_ data are usually normal or slightly high (typically FE_UA_; 5–15%) (Figure 3l), and they are predicted to have one or zero nonfunctional variants of *URAT1* or *GLUT9* (Figure 3m). Detection of one nonfunctional variant of *URAT1* (Figure 3n) or *GLUT9* (Figure 3o) by genetic analyses is necessary to make a diagnosis of RHUC type 1 (Figure 3q) or type 2 [3] (Figure 3r). If nonfunctional variants of *URAT1* or *GLUT9* are not detected (Figure 3p), physicians should consider differential diagnosis of RHUC [1] (Figure 3s). Thus, even before genetic tests of *URAT1* or *GLUT9,* FE_UA_ and SUA data are very helpful for the practical diagnosis of RHUC.

**Figure 4:**
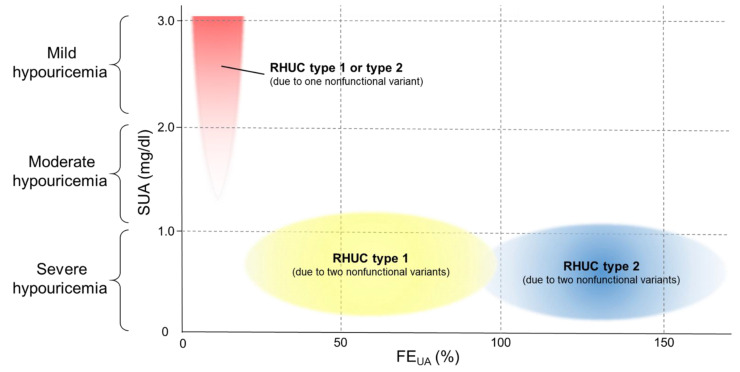
Specific distribution patterns of FE_UA_ and SUA of RHUC type 1 and 2 with the number of nonfunctional variants. This figure shows the relationship between RHUC and the number of nonfunctional variants of *URAT1* or *GLUT9* based on the patterns of FE_UA_ and SUA data. The horizontal and vertical axes respectively show FE_UA_ and SUA. Hypouricemic individuals (SUA ≤ 3.0 mg/dL) were divided into the following three groups: “severe hypouricemia” (SUA of ≤1.0 mg/dL), “moderate hypouricemia” (SUA of 1.1–2.0 mg/dL) and “mild hypouricemia” (SUA of 2.1–3.0 mg/dL). Typical laboratory data (FE_UA_ and SUA) for RHUC type 1 or 2 patients are shown in the following three patterns: The yellow and blue areas show “RHUC type 1 due to two nonfunctional variants of *URAT1*” (see also Figure 3h) and “RHUC type 2 due to two nonfunctional variants of *GLUT9*” (see also Figure 3i), respectively. The red area shows “RHUC type 1 due to one nonfunctional variant of *URAT1* (see also Figure 3q)” or “RHUC type 2 due to one nonfunctional variant of *GLUT9* (see also Figure 3r)”. Data from RHUC patients with other diseases, including renal dysfunction, might land in different areas from these three patterns. RHUC, renal hypouricemia; SUA, serum uric acid; FE_UA_, fractional excretion of uric acid.

Furthermore, Figure 4 illustrates the three specific distribution patterns of FE_UA_ and SUA data for RHUC, based on the number of nonfunctional variants of *URAT1* or *GLUT9*. The yellow area in Figure 4 (high FE_UA_ in severe hypouricemia, also see Figure 3c) shows the pattern for “RHUC type 1 due to two nonfunctional variants of *URAT1*”. The blue area in Figure 4 (extremely high FE_UA_ in severe hypouricemia, also see Figure 3d) shows the pattern for “RHUC type 2 due to two nonfunctional variants of *GLUT9*”. The red area in Figure 4 (normal or slightly high FE_UA_ in mild hypouricemia, also see Figure 3l) shows RHUC type 1 or RHUC type 2 due to one nonfunctional variant of *URAT1* or *GLUT9*. Interestingly, as shown in Figure 4, “RHUC due to two nonfunctional variants of *URAT1* or *GLUT9*” and “RHUC due to one nonfunctional variant of *URAT1* or *GLUT9*” were found to exhibit specific distribution patterns of FE_UA_ and SUA data. This suggests that it should be possible, even before genetic tests for *URAT1* or *GLUT9,* to predict the presence and number of nonfunctional variants of *URAT1* or *GLUT9* from the specific patterns shown in Figure 4. These three specific patterns of FE_UA_ and SUA data can also be useful for the selection of the appropriate genetic tests for *URAT1* or *GLUT9*, for the efficient and rapid diagnosis of RHUC.

As shown in Figure 3, Figure 4, there is an obvious difference between FE_UA_ levels of “RHUC type 1 due to two nonfunctional variants *URAT1*” and those of “RHUC type 2 due to two nonfunctional variants of *GLUT9*”. We consider one of the reasons for this difference to be as follows. While GLUT9 could be only one renal urate reabsorption transporter at the basolateral membrane in the human kidney, URAT1 is likely to play a role in urate handling alongside organic anion transporter 10 (OAT10/SLC22A13), the third and recently reported renal urate reabsorption transporter [24], at the apical membrane.

We believe that our findings will assist with a more practical diagnosis of RHUC based on the specific distribution patterns of FE_UA_ and SUA data. A more accurate diagnosis of RHUC will not only enable clinicians to prevent complications of RHUC such as EIAKI and urolithiasis, but will also lead us to a better understanding of the mechanism of urate handling and hypouricemia.

## 5. Conclusions

In summary, we have demonstrated four important findings. First, we investigated the prevalence of hypouricemia (SUA ≤ 2.0 mg/dL) and mild hypouricemia individuals (2.0 < SUA ≤ 3.0 mg/dL) among 30,685 Japanese participants, and discovered the prevalence of hypouricemic individuals (SUA ≤ 3.0 mg/dL) to be 0.97% in males and 6.94% in females. Second, we revealed a very high frequency of NFV-*URAT1* (W258X and R90H) in 1040 hypouricemia and mild hypouricemia individuals (SUA ≤ 3.0 mg/dL). Third, the presence and number of NFV-*URAT1* alleles assists with estimating the FE_UA_ data of hypouricemic individuals (SUA ≤ 3.0 mg/dL). This suggests that the FE_UA_ data of hypouricemic individuals should be very useful for predicting the presence and number of NFV-*URAT1* alleles, and also assists with diagnosis of RHUC type 1. Fourth, we were able to propose how to make a more reliable diagnosis of RHUC based on the distribution patterns of FE_UA_ and SUA data.

These findings have the potential to lead to a more practical diagnosis of RHUC based on specific patterns of laboratory data, and therefore a revision of the next edition of the clinical practice guideline for RHUC.

## Figures and Tables

**Table 1:** Distribution of SUA levels of 30,685 Japanese health examination participants.

SUA (mg/dL)	Male		Female	
	Number	Frequency (%)	Number	Frequency (%)
0.0–1.0	20	0.15	23	0.13
1.1–2.0	4	0.03	70	0.41
2.1–3.0	107	0.79	1093	6.40
3.1–7.0	10,716	78.75	15,703	91.95
7.1–8.0	1956	14.37	149	0.87
8.1–9.0	625	4.59	32	0.19
9.1–	179	1.32	8	0.05
Total	13,607	100	17,078	100

30,685 subjects (13,607 males and 17,078 females) were recruited from health examination participants at 5 collection sites for the Japan Multi-Institutional Collaborative Cohort Study (J-MICC Study). Frequency of hypouricemia (SUA of ≤2.0 mg/dL) was 0.18% (males) and 0.54% (females) among the 30,685 participants. Frequency of hypouricemic individuals (SUA of ≤3.0 mg/dL) was 0.97% (males) and 6.94% (females) among the 30,685 participants. SUA, serum uric acid.

**Table 2:** Characteristics of 1040 hypouricemic individuals.

	Male			Female		
	Number	Age (year)	BMI (kg/m^2^)	Number	Age (year)	BMI (kg/m^2^)
Severe hypouricemia (0.0–1.0 mg/dL)	17	56.4 ± 8.1	24.2 ± 2.6	19	55.7 ± 8.1	22.1 ± 2.9
Moderate hypouricemia (1.1–2.0 mg/dL)	4	53.5 ± 8.3	24.1 ± 2.1	57	50.2 ± 8.4	21.3 ± 3.1
Mild hypouricemia (2.1–3.0 mg/dL)	87	56.0 ± 8.9	22.6 ± 2.9	856	51.8 ± 9.2	21.1 ± 2.9
Hypouricemia (≤2.0 mg/dL)	21	55.8 ± 8.2	24.2 ± 2.5	76	51.6 ± 8.7	21.5 ± 3.1
Hypouricemia + mild hypouricemia (≤3.0 mg/dL)	108	56.0 ± 8.7	22.9 ± 2.9	932	51.8 ± 9.1	21.2 ± 2.9

See Figure 1 for the selection of 1040 hypouricemic individuals (SUA ≤ 3.0 mg/dL) from 30,685 Japanese health examination participants. Plus/minus values are means ± SD. BMI, body mass index; SUA, serum uric acid.

**Table 3:** The frequency of NFV-*URAT1* in 1040 hypouricemic individuals.

Hypouricemic Population (SUA)	Male				Female			
	Allele Number of NFV-*URAT1*			Total	Allele Number of NFV-*URAT1*			Total
	0	1	2		0	1	2	
Severe hypouricemia (0.0–1.0 mg/dL)	2 (11.8%)	4 (23.5%)	11 (64.7%)	17 (100%)	0 (0.0%)	6 (31.6%)	13 (68.4%)	19 (100%)
Moderate hypouricemia (1.1–2.0 mg/dL)	1 (25.0%)	3 (75.0%)	0 (0.0%)	4 (100%)	20 (35.1%)	37 (64.9%)	0 (0.0%)	57 (100%)
Mild hypouricemia (2.1–3.0 mg/dL)	29 (33.3%)	58 (66.7%)	0 (0.0%)	87 (100%)	570 (66.6%)	286 (33.4%)	0 (0.0%)	856 (100%)
Hypouricemia (≤2.0 mg/dL)	3 (14.3%)	7 (33.3%)	11 (52.4%)	21 (100%)	20 (26.3%)	43 (56.6%)	13(17.1%)	76 (100%)
Hypouricemia + mild hypouricemia (≤3.0 mg/dL)	32 (29.6%)	65 (60.2%)	11 (10.2%)	108 (100%)	590 (63.3%)	329 (35.3%)	13 (1.4%)	932 (100%)

See Figure 1 for the selection of 1040 hypouricemic individuals (SUA ≤ 3.0 mg/dL) from 30,685 Japanese health examination participants. W258X and R90H, the two most common variants of *URAT1*, were selected as NFV-*URAT1* in this study. NFV-*URAT1*, nonfunctional variants of *URAT1*; SUA, serum uric acid.

**Table 4:** Laboratory data and NFV-*URAT1* of 52 hypouricemic individuals.

Case No.	Sex	Age	NFV-*URAT1*		FE_UA_ (%)	SUA (mg/dL)	SCr (mg/dL)
			Number of Alleles	Amino Acid Substitution			
1	Female	69	2	W258X/W258X	51.32	0.5	0.6
2	Male	63	2	W258X/W258X	60.71	0.7	0.8
3	Female	68	2	W258X/W258X	40.55	0.8	0.7
4	Male	57	2	W258X/W258X	24.52	0.8	1.0
5	Female	45	1	W258X/	12.08	2.3	0.6
6	Female	56	1	W258X/	5.67	2.4	0.8
7	Male	69	1	W258X/	7.80	2.4	0.7
8	Female	61	1	W258X/	6.04	2.5	0.5
9	Female	51	1	W258X/	6.40	2.6	0.6
10	Female	70	1	W258X/	6.97	2.6	0.6
11	Female	68	1	W258X/	2.17	2.8	0.6
12	Female	55	1	W258X/	10.41	2.9	0.7
13	Female	41	1	W258X/	11.12	2.9	0.4
14	Male	69	1	W258X/	12.51	3.0	0.9
15	Male	55	1	W258X/	8.19	3.0	0.9
16	Female	46	1	R90H/	6.55	3.0	0.5
17	Female	65	0		12.45	2.0	0.5
18	Male	54	0		4.07	2.3	0.6
19	Female	61	0		14.75	2.3	0.5
20	Female	62	0		3.40	2.5	0.5
21	Female	45	0		3.10	2.6	0.6
22	Female	71	0		12.32	2.6	0.6
23	Male	52	0		4.91	2.6	0.7
24	Female	50	0		2.94	2.7	0.6
25	Female	54	0		6.16	2.7	0.6
26	Female	52	0		2.05	2.7	0.6
27	Female	62	0		9.51	2.8	0.5
28	Female	41	0		7.86	2.8	0.6
29	Female	62	0		11.76	2.8	0.5
30	Female	58	0		12.11	2.8	0.6
31	Female	47	0		6.77	2.8	0.7
32	Female	43	0		8.11	2.8	0.5
33	Female	41	0		8.17	2.8	0.6
34	Female	60	0		5.88	2.8	0.7
35	Male	59	0		16.86	2.8	0.8
36	Female	51	0		5.27	2.8	0.7
37	Female	58	0		12.61	2.9	0.5
38	Female	43	0		6.64	2.9	0.7
39	Female	47	0		7.90	2.9	0.6
40	Female	68	0		8.14	2.9	0.7
41	Female	72	0		14.10	2.9	0.4
42	Female	52	0		5.91	3.0	0.6
43	Female	63	0		6.07	3.0	0.5
44	Female	51	0		8.29	3.0	0.5
45	Female	69	0		8.17	3.0	0.5
46	Female	47	0		8.15	3.0	0.6
47	Female	74	0		3.34	3.0	0.6
48	Female	59	0		10.43	3.0	0.6
49	Female	50	0		8.68	3.0	0.6
50	Female	60	0		9.96	3.0	0.6
51	Female	56	0		7.68	3.0	0.6
52	Female	64	0		4.17	3.0	0.6

Fifty-two hypouricemic individuals (SUA ≤ 3.0 mg/dL) were found among 2240 individuals whose FE_UA_ data were available. These 52 hypouricemic individuals include 4 hypouricemia cases (SUA ≤ 2.0 mg/dL; 2 males and 2 females) with 2 alleles of NFV-*URAT1*, and 47 mild hypouricemia cases (2.0 < SUA ≤ 3.0 mg/dL; 6 males and 41 females) with 1 or 0 alleles of NFV-*URAT1*. Four hypouricemia cases with two alleles of NFV-*URAT1* (Case Nos. 1–4) exhibit severe hypouricemia (SUA ≤ 1.0 mg/dL), and the average of their FE_UA_ was 44.3% (range: 24.5–60.7%). On the other hand, the average of FE_UA_ for 12 mild hypouricemia with only 1 allele of NFV-*URAT1* (Case Nos. 5–16) was 7.99% (range: 2.17–12.51%). Case No. 17 (hypouricemia) exhibits an SUA of 2.0 mg/dL and FE_UA_ of 12.45%, suggesting this individual to also be a renal hypouricemia (RHUC) case with a different (but only one) nonfunctional variant of *URAT1/SLC22A12* (or *GLUT9/SLC2A9*). Case Nos. 18–52 had mild hypouricemia, showing an SUA of 2.1–3.0 mg/dL and FE_UA_ of 2.05–16.86%, values very similar to those of cases Nos. 5–16 who have one NFV-*URAT1* allele. Therefore, case Nos. 18–52 appear to have a different (but only one) nonfunctional variant of *URAT1/SLC22A12* (or *GLUT9/SLC2A9*). W258X and R90H, the two most common variants of *URAT1*, were selected as NFV-*URAT1* in this study. NFV-*URAT1*, nonfunctional variants of *URAT1*; FE_UA_, fractional excretion of uric acid; SUA, serum uric acid; SCr, serum creatinine.

**Table 5:** Multiple regression analysis of FE_UA_ and SUA along the number of NFV-*URAT1*.

		Male		Female	
		Partial Regression Coefficient	*p* Value	Partial Regression Coefficient	*p* Value
**FE_UA_**	β_0_	3.94	0	5.40	1.61 × 10^−249^
	β_1_	2.63	4.04 × 10^−20^	―	―
	β_2_	38.68	1.35 × 10^−108^	40.54	2.15 × 10^−79^
**SUA**	β_0_	6.10	0	4.56	0
	β_1_	–1.93	6.56 × 10^−45^	−1.25	1.53 × 10^−7^
	β_2_	–5.35	2.39 × 10^−12^	−3.91	2.97 × 10^−8^

2240 individuals (1542 males and 698 females) with their FE_UA_ data available were analyzed. y = β_0_ +  β_1_x_1_  + β_2_x_2_, where y is FE_UA_ or SUA levels, x_1_ is a dummy variable representing whether the number of alleles on NFV-*URAT1* is one (one allele = 1 and other = 0) and x_2_ is a dummy variable representing whether the number of alleles on NFV-*URAT1* is two (two alleles = 1 and other = 0). W258X and R90H, the two most common variants of *URAT1*, were selected as NFV-*URAT1* in this study. ―, eliminated from covariance. FE_UA_, fractional excretion of uric acid; SUA, serum uric acid; NFV-*URAT1*, nonfunctional variants of *URAT1*.

## Data Availability

The data presented in this study are available upon request from the corresponding author.

## References

[1] Nakayama A., Matsuo H., Ohtahara A., Ogino K., Hakoda M., Hamada T., Hosoyamada M., Yamaguchi S., Hisatome I., Ichida K. (2019). Clinical practice guideline for renal hypouricemia (1st edition). Hum. Cell.

[2] Enomoto A., Kimura H., Chairoungdua A., Shigeta Y., Jutabha P., Cha S.H., Hosoyamada M., Takeda M., Sekine T., Igarashi T. (2002). Molecular identification of a renal urate anion exchanger that regulates blood urate levels. Nature.

[3] Matsuo H., Chiba T., Nagamori S., Nakayama A., Domoto H., Phetdee K., Wiriyasermkul P., Kikuchi Y., Oda T., Nishiyama J. (2008). Mutations in glucose transporter 9 gene *SLC2A9* cause renal hypouricemia. Am. J. Hum. Genet..

[4] Ishikawa I. (2002). Acute renal failure with severe loin pain and patchy renal ischemia after anaerobic exercise in patients with or without renal hypouricemia. Nephron.

[5] Kikuchi Y., Koga H., Yasutomo Y., Kawabata Y., Shimizu E., Naruse M., Kiyama S., Nonoguchi H., Tomita K., Sasatomi Y. (2000). Patients with renal hypouricemia with exercise-induced acute renal failure and chronic renal dysfunction. Clin. Nephrol..

[6] Ichida K., Hosoyamada M., Hisatome I., Enomoto A., Hikita M., Endou H., Hosoya T. (2004). Clinical and molecular analysis of patients with renal hypouricemia in Japan-influence of *URAT1* gene on urinary urate excretion. J. Am. Soc. Nephrol..

[7] Sakiyama M., Matsuo H., Shimizu S., Nakashima H., Nakamura T., Nakayama A., Higashino T., Naito M., Suma S., Hishida A. (2016). The effects of *URAT1/SLC22A12* nonfunctional variants, R90H and W258X, on serum uric acid levels and gout/hyperuricemia progression. Sci. Rep..

[8] Wakasugi M., Kazama J.J., Narita I., Konta T., Fujimoto S., Iseki K., Moriyama T., Yamagata K., Tsuruya K., Asahi K. (2015). Association between hypouricemia and reduced kidney function: A cross-sectional population-based study in Japan. Am. J. Nephrol..

[9] Kuwabara M., Niwa K., Ohtahara A., Hamada T., Miyazaki S., Mizuta E., Ogino K., Hisatome I. (2017). Prevalence and complications of hypouricemia in a general population: A large-scale cross-sectional study in Japan. PLoS ONE.

[10] Kawasoe S., Ide K., Usui T., Kubozono T., Yoshifuku S., Miyahara H., Maenohara S., Ohishi M., Kawakami K. (2019). Distribution and Characteristics of Hypouricemia within the Japanese General Population: A Cross-Sectional Study. Medicina.

[11] Asai Y., Naito M., Suzuki M., Tomoda A., Kuwabara M., Fukada Y., Okamoto A., Oishi S., Ikeda K., Nakamura T. (2009). Baseline data of Shizuoka area in the Japan Multi-Institutional Collaborative Cohort Study (J-MICC Study). Nagoya J. Med. Sci..

[12] Hamajima N., Group J.-M.S. (2007). The Japan Multi-Institutional Collaborative Cohort Study (J-MICC Study) to detect gene-environment interactions for cancer. Asian Pac. J. Cancer Prev..

[13] Ichida K., Matsuo H., Takada T., Nakayama A., Murakami K., Shimizu T., Yamanashi Y., Kasuga H., Nakashima H., Nakamura T. (2012). Decreased extra-renal urate excretion is a common cause of hyperuricemia. Nat. Commun..

[14] Gabrikova D., Bernasovska J., Sokolova J., Stiburkova B. (2015). High frequency of SLC22A12 variants causing renal hypouricemia 1 in the Czech and Slovak Roma population; simple and rapid detection method by allele-specific polymerase chain reaction. Urolithiasis.

[15] Stiburkova B., Gabrikova D., Cepek P., Simek P., Kristian P., Cordoba-Lanus E., Claverie-Martin F. (2016). Prevalence of *URAT1* allelic variants in the Roma population. Nucleosides Nucleotides Nucleic Acids.

[16] Nakayama A., Matsuo H., Abhishek A., Ichida K., Shinomiya N. (2021). Guideline Development Committee of Clinical Practice Guideline for Renal Hypouricemia., First clinical practice guideline for renal hypouricemia: A rare disorder that aided the development of urate-lowering drugs for gout. Rheumatology.

[17] Ichida K., Hosoyamada M., Kamatani N., Kamitsuji S., Hisatome I., Shibasaki T., Hosoya T. (2008). Age and origin of the G774A mutation in *SLC22A12* causing renal hypouricemia in Japanese. Clin. Genet..

[18] Wakida N., Tuyen D.G., Adachi M., Miyoshi T., Nonoguchi H., Oka T., Ueda O., Tazawa M., Kurihara S., Yoneta Y. (2005). Mutations in human urate transporter 1 gene in presecretory reabsorption defect type of familial renal hypouricemia. J. Clin. Endocrinol. Metab..

[19] Toyoda Y., Kawamura Y., Nakayama A., Nakaoka H., Higashino T., Shimizu S., Ooyama H., Morimoto K., Uchida N., Shigesawa R. (2021). Substantial anti-gout effect conferred by common and rare dysfunctional variants of *URAT1/SLC22A12*. Rheumatology.

[20] Kawamura Y., Toyoda Y., Ohnishi T., Hisatomi R., Higashino T., Nakayama A., Shimizu S., Yanagi M., Kamimaki I., Fujimaru R. (2020). Identification of a dysfunctional splicing mutation in the *SLC22A12/URAT1* gene causing renal hypouricaemia type 1: A report on two families. Rheumatology.

[21] Dinour D., Gray N.K., Campbell S., Shu X., Sawyer L., Richardson W., Rechavi G., Amariglio N., Ganon L., Sela B.A. (2010). Homozygous *SLC2A9* mutations cause severe renal hypouricemia. J. Am. Soc. Nephrol..

[22] Dinour D., Gray N.K., Ganon L., Knox A.J., Shalev H., Sela B.A., Campbell S., Sawyer L., Shu X., Valsamidou E. (2012). Two novel homozygous *SLC2A9* mutations cause renal hypouricemia type 2. Nephrol. Dial. Transplant..

[23] Ichida K., Amaya Y., Okamoto K., Nishino T. (2012). Mutations associated with functional disorder of xanthine oxidoreductase and hereditary xanthinuria in humans. Int. J. Mol. Sci..

[24] Higashino T., Morimoto K., Nakaoka H., Toyoda Y., Kawamura Y., Shimizu S., Nakamura T., Hosomichi K., Nakayama A., Ooyama K. (2020). Dysfunctional missense variant of *OAT10/SLC22A13* decreases gout risk and serum uric acid levels. Ann. Rheum. Dis..

